# Supporting Breastfeeding in 2021 and Beyond—Lessons from the Pandemic

**DOI:** 10.3390/pediatric13020037

**Published:** 2021-06-01

**Authors:** Ajay Pratap Singh, Vasantha HS Kumar, Sanjeet Panda

**Affiliations:** 1 Paul L. Foster School of Medicine, Texas Tech University Health Sciences Center, El Paso, TX 79905, USA; Sanjeet.Panda@ttuhsc.edu; 2 Department of Pediatrics, University at Buffalo, Oishei Children’s Hospital, Buffalo, NY 14203, USA; vkumar3@buffalo.edu

**Keywords:** supporting breastfeeding, breastfeeding during pandemic, paid parental leave, breastfeeding future, lactation support, telelactation

## Abstract

The COVID-19 pandemic has affected maternal and infant health globally both directly from infection with the SARS-CoV-2 virus and indirectly from changes in health care resulting from social, economic, and health care policies unique to each country. The developing countries have to share the disproportionate burden on maternal and infant health. In this review, we discuss the uncertainties resulting from SARS-CoV-2 infection in pregnancy, vertical transmission of the virus, and its effects on breastfeeding of the newborn. The problems of families and communities caring for mothers with COVID-19 and its impact on breastfeeding in newborns are discussed. The challenges posed by the pandemic have forced us to think and devise innovative solutions, including telemedicine help for antenatal counseling, breastfeeding education, and lactation support. Optimal utilization of resources and technology to find creative solutions at the individual and the community level will help in facilitating maternal–infant bonding soon after birth. Appropriate health care policies to support pregnant and lactating mothers will go a long way in meeting healthy child development goals.

## 1. Introduction

The coronavirus pandemic has affected every aspect of life and has virtually changed how humans lead their day-to-day lives [1]. Since the onset of the pandemic, most countries, including the United States, have implemented restrictions on daily activities in some shape or form. Becoming a parent is challenging at the best of times. The COVID-19 (coronavirus illness of 2019) pandemic and the subsequent lockdown to prevent the spread of severe acute respiratory syndrome coronavirus 2 (SARS-CoV-2) virus have placed an additional burden on families. One area that has been affected immensely is the delivery of health care, and, in that, a significant subsection of breastfeeding mothers has probably suffered equally [2]. As we navigate this forever-changed world, we need to reflect on the lessons being learned and how to capitulate on positive things and explore new frontiers to support breastfeeding in the year 2021 and beyond. Here, we present a review of the relevant effects of a pandemic on breastfeeding and present ideas to promote breastfeeding (Table 1).

## 2. COVID Pandemic and Maternal–Infant Health

There are only a few infectious contraindications to breastfeeding or the provision of expressed breastmilk, except human immunodeficiency virus (HIV) in industrialized nations, human T cell lymphotropic virus, active Ebola infection, and untreated brucellosis [3,4,5]. Time after time, history has shown how resilient and protected breastmilk formation is against almost all infectious agents. With nearly one year into the pandemic, we now know with near certainty that the same is true about the transmission of the SARS-CoV-2 virus [4,6,7].

The financial benefits of optimal breastfeeding rates to the United States economy are in tens of billions of dollars [8,9]. Any decline in breastfeeding rates leads to unnecessary expenditure to the tune of up to 3.7 billion dollars on formulas [9]. We learned very little from previous viral outbreaks, including SARS-CoV-1 in 2003 (10), H1N1 flu in 2009 (11), and now SARS-CoV-2, by being reactive in our approach. Meaning we have a viral outbreak, we try to contain it, then sometimes later, the effects of that virus on human milk come to be known. We need to change our approach to a more proactive virus surveillance research [10] with breastmilk research. In the future, in the event of a viral outbreak or any other potential threats, it can be known ahead of time if breastfeeding or breastmilk provision is safe.

### 2.1. Uncertainties over Vertical Transmission of SARS-CoV-2

Questions regarding vertical transmission, including breastfeeding safety by mothers infected with SARS-CoV-2, are of great importance. During the early days of the pandemic, lack of information regarding vertical transmission of SARS-CoV-2 in breast milk led to the rapid dissemination of misinformation that newborns carry a higher risk of SARS-CoV-2 from infected mothers. The risk of infection led to additional hurdles that made the smooth transition to providing breast milk and breastfeeding that much more difficult during the early stages of the pandemic.

The option of COVID-19 vertical transmission has been examined in several studies. A systematic review of 11 studies did not demonstrate any intrauterine or transplacental transmission of the SARS-CoV-2 virus to the fetus during the 3rd trimester of pregnancy [11]. Several studies have suggested that vertical transmission is possible from mother to infant, based on IgM antibodies [12,13] and SARS-CoV-2 viral RNA isolated from blood and nasopharyngeal swabs [13,14]. Studies have shown that despite strict infection control and prevention measures during the delivery process, the upper respiratory tract in the newborns was positive for the SARS-CoV-2 virus indicating the virus was maternal in origin [15,16]. Transplacental transfer of SARS-CoV-2 in a neonate born to a mother who became infected with the virus in the third trimester has been reported [17].

Defining vertical transmission based on isolated case reports is difficult due to a lack of standardization regarding the timing and the type of specimen collected. In a large study of 101 newborns born to COVID-positive mothers, no evidence of perinatal transmission was observed, despite two infants having results demonstrating low viral loads [18]. Current evidence suggests that it is probable that perinatal transmission could occur in 1–2% of neonates born to mothers with SARS-CoV-2 infection. However, the likelihood of perinatal transmission is very low [19].

At present, it is difficult to conclude from case reports whether the resultant infections occurred from horizontal transmission at birth versus vertical transmission through the placenta. The expression of angiotensin-converting enzyme 2 (ACE2), the receptor for SARS-CoV-2 virus, on the maternal–fetal interface cells, such as the stromal cells, cytotrophoblasts, and syncytiotrophoblasts, on the placenta and other fetal organs [20] may suggest that vertical transmission is possible and needs further investigation. Although the weight of the evidence demonstrates the lack of inutero transmission of the SARS-CoV-2 virus, the possibility of transplacental transmission of SARS-CoV-2 is unclear at present. However, a growing body of evidence demonstrates an association between SARS-CoV-2 infection during pregnancy and preterm delivery [21]. Maternal SARS-CoV-2 infection in pregnancy is associated with only a slight increase in the absolute risk of neonatal morbidities with no increase in neonatal mortality [22].

### 2.2. Breastfeeding and COVID-19 Infection

Human milk, including colostrum, provides complete nutrition to the newborn infant and provides natural bioactive factors, antibodies, and targeted immunologic mediators that protect the infant from the viral disease [23,24]. The initial guidelines issued by the AAP at the onset of the pandemic in 2020 reflected the thinking that SARS-CoV-2 is a contagious infection and infected individuals would experience severe morbidity and mortality [7]. The guidelines were cautious and conservative due to the lack of data on the disease’s evolution, particularly in vertical transmission and breastfeeding. The guidelines included strict PPE, separation of infants born to infected mothers from uninfected newborns until their status is established, negative pressure rooms for infants under investigations, and N95 masks for providers. Mothers who request direct breastfeeding should comply with the strict preventive precautions that include the use of a mask and meticulous breast and hand hygiene. Mothers may express breast milk after appropriate breast and hand hygiene. Caregivers who are not infected may feed the breast milk to the infant [7].

The guidelines created confusion and fear in the public and the professionals involved regarding breastfeeding of asymptomatic newborns. Breastfeeding took a back seat at this time. However, the AAP has been serially updating COVID-19 guidelines with the rapidly emerging evidence that has informed the epidemiology, immunology, and clinical implications of maternal SARS-CoV-2 infection at the time of delivery. The most recent guidelines strongly support breastfeeding as the best choice for infant feeding, even if the mother or her infant is infected with SARS-CoV-2. AAP recommends rooming-in of infants with the mothers per hospital practices unless the mother is too ill to care for their infant. The mother should perform appropriate infection-control precautions, such as hand hygiene, and wear a mask during breastfeeding and care of the newborn infant. For mothers who choose not to breastfeed, they may express breastmilk after appropriate hand hygiene. The guidelines have moved towards being more friendly for breastfeeding and mother–infant bonding. Maternal–newborn rooming-in with infection control precautions appeared to be effective in preventing newborn disease transmission [25]. For infants infected with the SARS-CoV-2 virus, the parents should be encouraged to visit their infant in full PPE with airborne precautions until the infant is taken off COVID precautions.

### 2.3. Breast Milk and Antibodies to SARS-CoV-2

PCR-based detection of the SARS-CoV-2 virus has been detected in breast milk in several case reports [26,27]. Several large studies have demonstrated the lack of maternal-to-child transmission of the SARS-CoV-2 virus via breast milk [28,29]. It is important to note that the milk produced by infected mothers is a source of anti-SARS-CoV-2 IgA and IgG antibodies and neutralizes SARS-CoV-2 activity [29]. The downside of PCR diagnostic tests is they detect the viral nucleic acid for up to 12 weeks, even after the person is no longer infectious [30]. Detection of viral RNA by RT-PCR does not necessarily mean infectivity of the patient. In a large study of 18 asymptomatically infected women, the authors could not culture live virus from breast milk [28]. Studies had also demonstrated that Holder pasteurization kills live viruses when experimentally added to breast milk, suggesting that the process of preparing donor milk could eliminate infectious viruses if present [28,31]. With COVID-19 precaution measures, it is appropriate to provide either fresh breast milk or donor milk for sick infants in the NICU. The results support the recommendations to continue breastfeeding during mild-to-moderate maternal COVID-19 illness.

### 2.4. COVID-19 and Kangaroo Mother Care

Kangaroo mother care (KMC), which involves prolonged skin-to-skin contact and promotion of exclusive breastfeeding, is one of the most inexpensive methods of promoting early discharge, particularly in developing countries. In the latest Cochrane review, infants who received KMC had a substantial reduction in mortality, hypothermia, and severe infections compared to standard care [32]. COVID-19 has disrupted health services for mothers and newborns, particularly in low- and middle-income countries. A recent study has demonstrated the survival benefit of KMC in preterm infants <2000 g birth weight that far outweighs the small risk of death due to COVID-19 in neonates. An estimated 50% reduction in KMC coverage could result in 12,570 total deaths, and a complete disruption could result in 25,140 incremental deaths, representing a 2.3–4.6% increase in neonatal mortality across 127 countries [33]. The study quantifies the potential impact on neonatal survival of KMC compared to coverage disruptions due to COVID-19. KMC and breastfeeding should be encouraged for all mothers and newborns, including mothers with confirmed or suspected COVID-19. If mothers are unwell, healthy family members may provide KMC. The post-pandemic recovery period presents opportunities to rebuild and invest in achieving universal coverage of high-quality maternal and child health services, including KMC [33].

### 2.5. Uncertainties Regarding Discharge Planning and Homecare

During the early stages of the pandemic, separation of babies from infected mothers or mothers under investigation for COVID placed a burden on breastfeeding. Continued separation of the mother and the infant until the guidelines became clearer put additional hurdles on breastfeeding and care of the newborn after discharge. Even though SARS-CoV-2 nucleic acid is detected in breast milk, the infectious virus’ presence has not been reported. Post-discharge guidance and education are essential to support families, ensure the mother’s and infant’s health, and facilitate breastfeeding.

Although the breastfeeding effects on SARS-CoV-2 infection are not known, natural protective factors in breast milk and its ability to protect against viral infections are enormous benefits, especially during the pandemic. In the age of lockdown, with the potential for shortages of formula and its supplies, breastfeeding offers a sustainable mode of nutrition to infants. Mothers should be encouraged to breastfeed the infant with a mask and proper hand hygiene. Mothers who are positive for SARS-CoV-2 still have to maintain a safe distance from the infant whenever possible and use a mask and hand hygiene during direct contact with the infant until she is afebrile for 24 h or symptom-free for ten days. Despite the best of the circumstances, barriers placed for the infant’s and mother’s benefit may make the mother not breastfeed during the first few weeks after birth. However, mothers who intend to breastfeed can take the help of galactagogues, if required, which may help in establishing lactation and breastfeeding [34].

### 2.6. Uncertainties Regarding Vaccination of Pregnant and Lactating Mothers

The first coronavirus-19 (COVID-19) vaccines available in the United States were messenger RNA (mRNA)-based vaccines by Pfizer (BNT162b2) and Moderna (mRNA-1273). In December 2020, the U.S. Food and Drug Administration (FDA) issued an Emergency Use Authorization (EUA) for both the Pfizer (two-dose series, 3 weeks apart) and the Moderna (two-dose series, one month apart) vaccines as recommended by the Advisory Committee on Immunization Practices (ACIP). The mRNA-based vaccines are relatively expensive and are mostly available in developed countries at the current time. The AstraZeneca and the Janssen Biotech Inc. (Johnson & Johnson) vaccines are based on the human adenovirus technology platform, relatively inexpensive and most suitable for developing countries. The vaccines available for the SARS-CoV-2 virus are not live virus vaccines and encode for a stable form of acute respiratory syndrome coronavirus-2 (SARS-CoV-2) spike protein. The pace of vaccine production is not keeping up with the spread of SARS-CoV-2 infection, resulting in severe disease and death worldwide. Irrespective of the type of vaccines, all countries should collaborate and act quickly to control the spread of SARS-CoV-2 infection and prevent COVID-19. The second wave of COVID-19 disease in India is a case in point about the dangerous nature of the SARS-CoV-2 illness and the resultant pandemic.

Pregnant women were excluded from the initial trials, and limited data were available at the time of initial authorization of the vaccines. Pregnant women were significantly more likely than were nonpregnant women to be admitted to an intensive care unit (ICU), receive invasive ventilation and extracorporeal membrane oxygenation (ECMO) [35]. Pre-existing comorbidities, high maternal age, and increased body mass index seem to be risk factors for severe COVID-19. Preterm birth rates are higher in pregnant women with COVID-19 than in pregnant women without the disease [36]. Early data from the v-safe surveillance system, the v-safe pregnancy registry, and the Vaccine Adverse Event Reporting System (VAERS) do not indicate any obvious safety signals concerning pregnancy or neonatal outcomes associated with COVID-19 vaccination in the third trimester of pregnancy [37].

Historically, vaccine development has suffered from delayed or no participation of pregnant or breastfeeding mothers in clinical trials due to ethical–legal fears and fear of effects on the developing fetus and breastfeeding infants [38,39]. The Centers for Disease Control and Prevention (CDC) and ACIP, in collaboration with the American College of Obstetricians and Gynecologists (ACOG) and the American Academy of Pediatrics (AAP), have issued guidance indicating that COVID-19 vaccines should not be withheld from pregnant persons [40,41,42]. Although data on the effects of the COVID vaccine on expectant mothers and during lactation are scarce, they accumulate at a fast clip. A conversation between the patient and their clinical team may assist with informed decision-making regarding the use of vaccines approved under EUA to prevent COVID-19 in pregnant patients. Importantly, pregnancy testing should not be a requirement before receiving any EUA-approved COVID-19 vaccines. Unfounded claims linking COVID-19 vaccines to infertility have been scientifically disproven. ACOG recommends vaccination for all eligible people who may consider future pregnancy [40]. Both the providers and the mother need to get information from reliable sources such as the CDC, ACOG, and the AAP [40,41,42]. Guidance from the above organizations would go a long way in presenting clarity and a unified approach regarding vaccination in pregnant and lactating mothers.

### 2.7. Comprehensive National Paid Maternity Leave Policy to Promote Breastfeeding

Paid maternity leave is shown to improve mental and physical health, decrease rehospitalizations and mortality of infants and mothers, and increase the initiation and duration of breastfeeding. Employed women who received 12 or more weeks of paid maternity leave were more likely to initiate breastfeeding and be breastfeeding their child at six months than those without paid leave [43]. In a systemic review [44] from Organization for Economic Co-operation and Development (OECD) countries, some form of national paid leave policy at the federal level had no adverse effect on the overall economy and revealed consistent improvements in infant and child health. Extending the duration of legislated paid maternity leave appears to promote breastfeeding practices in low- and middle-income countries and a potential mechanism to reduce barriers to breastfeeding for working mothers [45].

The massive layoff and the rise in unemployment by the coronavirus have affected women especially hard, according to the U.S. Bureau of Labor Statistics [46]. As per the U.S. National Bureau of Economic Research [47], about 1 in every four women in the U.S. alone reported becoming unemployed in the year 2020; most women said childcare as the reason was twice the rate as men. Currently, only five states in the U.S. have laws in place providing some form of paid leaves to new parents [48]. California, Rhode Island, Washington State, New Jersey, and New York are the states that provide paid leave, New York being the only state to provide 12 weeks of maternity leave with pay. The absence of paid maternity leave in most states in the United States is acutely felt in the pandemic, exacerbating breastfeeding problems, childcare issues, and psychosocial stress. The list does not even include the effects of economic poverty on vital matters such as family stability and maternal and infant health. The pandemic has shown that it is only befitting for the U.S. to join the rest of the developed world in providing paid parental leave across the nation. Our children are the national asset, and it is only appropriate to care for them in crisis to help them and the country in preparing for the future.

The 2011 U.S. general’s call to action to support breastfeeding identified several of these barriers [49] that included adopting breastfeeding-friendly legislation at each state level. Even though the U.S. General’s report on supporting breastfeeding is impressive, the pandemic has shown that the U.S. needs a unified approach in dealing with maternal and infant issues, including breastfeeding across the nation. A lactating mother is left to navigate the maze of state-supported legislation. Many of the laws lack enforcement power, and only a few include penalties for violations, making them significantly less effective. The pandemic has exposed the U.S. healthcare system’s deficiencies, especially relating to maternal and infant health. Addressing these issues now on a war footing would help the nation to be better prepared for the future.

### 2.8. Maternal Health and Breastfeeding

The COVID-19 pandemic has resulted in substantial social and economic challenges, impacting mental health during pregnancy and in the perinatal period. The prevalence of maternal depression and anxiety symptoms among mothers in a Canadian cohort increased during the COVID-19 pandemic. The increases were related to income disruptions, difficulty balancing homeschooling with work responsibilities, and difficulties in obtaining childcare [50]. The COVID-19 pandemic has negatively impacted mothers’ mental health status, worsening prenatal care delivery and threatening the well-being of the fetus and the newborn [51,52]. Untreated maternal depression has multiple adverse effects on maternal–infant bonding, breastfeeding, and child development. Psychotherapeutic interventions of postpartum depression, especially during the pandemic, could positively promote breastfeeding, parenting, and the health of both the mother and the infant [53]. Support from the relevant agencies to help stabilize financial security, increasing job opportunities, availability of childcare, and increased social support will be crucial to support maternal mental health and favorable long-term outcomes in children.

## 3. Innovative Solutions to Support Breastfeeding

### 3.1. Telemedicine Support for Promoting Maternal Health and Breastfeeding

There has been a technological revolution in new ways of conducting healthcare, including the advancement and near-total acceptance of telemedicine across the nation. The rise of telemedicine has been the silver lining on the pandemic in the management of patients. Advances in wearable devices for vital sign monitoring, ubiquitous availability of smartphones, and requisite internet infrastructure at both sides of the clinical encounter have facilitated telemedicine and accelerated by the pandemic [54]. Telemedicine can help in the treatment of mildly ill patients, minimizing exposures and preventing the spread of the disease [55]. Hospitals invested in telemedicine are well-positioned to ensure that the patients with COVID-19 receive the care they need. Telemedicine may be the perfect solution to deal with the highly infectious nature of SARS-CoV-2 and its hospital-related transmission [56].

Telehealth lifestyle interventions are gaining popularity for use in pregnancy for the management of complications such as gestational diabetes [57], monitoring of blood pressure [58], and preventing excess gestational weight gain [59]. Telehealth has also been used in low- and middle-income countries, particularly in rural communities where access to antenatal care is challenging [60]. Telemedicine has been shown to improve prenatal care quality by better observing pregnant women [61]. Incorporating some of these platforms will go a long way in overcoming hurdles in delivering health care to infants and mothers. Telemedicine could facilitate successful sessions that include relaxation techniques, problem-solving, and cognitive-based therapies in managing postpartum depression, especially in times of the pandemic [62].

### 3.2. NICU Visitation Policy and COVID-19

The implementation of family-centered care has increased in recent years due to many health benefits for neonates and families. The coronavirus pandemic has resulted in restrictions on visitation to the Neonatal Intensive Care Unit (NICU). Hospital restrictions have a significantly limited parental presence in the NICU, although single-family room design attenuates this effect [63]. Protocols, particularly regarding visitation by symptomatic and asymptomatic mothers, should be built to facilitate mother–infant bonding and promote breastfeeding. Helping parents cope with the stressful NICU experiences has a significant impact on parents and newborns alike. All mothers of infants admitted to NICU feel separated from their young ones [30,31]; this was true before the pandemic. The ongoing pandemic exaggerates the effect due to visitation restrictions and lockdowns. Innovative solutions to decrease maternal stress and anxiety that could disrupt breast milk supply are of critical importance. Visitation restriction could be overcome by web-based video conferencing and extensive use of smartphone applications (such as facetime or skype) to communicate and bond with the infant, remove barriers and help to connect with their infant [64]. The adoption of round-the-clock secure live video streaming may improve care by enhancing parental involvement in their infant’s ongoing care [65,66]. The ability to see one’s baby anytime not only decreases maternal anxiety and stress but also provides a feeling of closeness and fulfillment, facilitating breastmilk production. Kangaroo mother care (KMC) is another effective solution in the initiation of exclusive breastfeeding. The current recommendations from the WHO are to not separate mothers from their newborns and encourage the practice of KMC in cases of suspected or confirmed COVID-19 by using personal protective equipment and disinfection of used surfaces [67].

### 3.3. Contactless Delivery of Breast Milk

**Figure 1:**
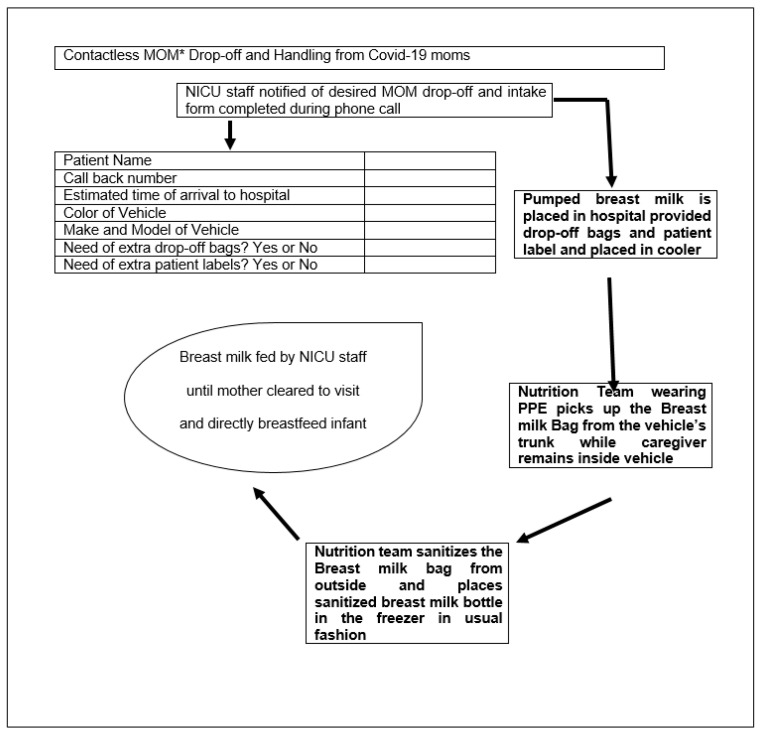
Contactless Mother’s Own Milk (*MOM) Drop-Off Process at El Paso Children Hospital Neonatal intensive care unit at Texas Tech University and Health Sciences Center, El Paso, Texas, USA.

The feeding of human milk to preterm infants is typically much more complicated than the mere act of breastfeeding. Mother’s milk must be pumped, labeled, transported to the hospital, stored, tracked for appropriate expiration dates and times, thawed (if previously frozen), fortified, and administered to the infant. Care should be taken at each step of the process to avoid microbial contamination, misadministration (the wrong milk for the wrong patient), fortification errors, and waste [68]. Staff should be well trained in aseptic techniques and demonstrate proper steps for handling human milk and fortifiers. Hand hygiene is critical in handling human milk to prevent the introduction of exogenous microbial contamination, especially in times of COVID-19 pandemic [68]. Refrigeration guidelines for the storage of human milk for healthy infants at home have been described [69]. Storage times and temperatures impact nutritional quality, biologically active components in human milk, and rate/incidence of microbial growth [70]. Hospital systems should develop a strategy for breastmilk delivery from the quarantining mother to her hospitalized infant. Attempts have been made to promote contactless healthcare delivery through telemedicine, remote monitoring of patients, and chronic management of diseases [71,72]. A model of contactless delivery of breast milk from mothers to infants practiced at Texas Tech University Health Science Center, El Paso Children’s Hospital NICU, Texas, is illustrated in Figure 1. Every healthcare system should strive to develop and support low-cost strategies that suit their unique situation.

### 3.4. Telelactation

Connecting with lactation specialists over Telehealth requires planning and proactive participation by both providers and parents to seamless service integration into the clinical domain. Telelactation is an innovation in the delivery of professional breastfeeding support. Telehealth services connecting breastfeeding mothers to remotely located lactation consultants have been shown to support breastfeeding in an underserved population [73]. Telehealth visits should encompass all aspects of breastfeeding, including assessment, breast milk transfer, and weight checks.

### 3.5. Antenatal Counseling and Breastfeeding Education

The pandemic has upended the current way of providing prenatal and postpartum education, and has introduced unforeseen challenges in the delivery of lactation training, education, and skilled support worldwide. Barriers to breastfeeding also include the lack of in-person support and education to mothers during the prenatal and postnatal period [2,74]. *Ready, Set, BABY* (RSB) is an open access prenatal breastfeeding curriculum created by the Carolina Global Breastfeeding Institute to provide virtual prenatal breastfeeding education during the COVID-19 pandemic [75]. Despite the rapid adoption of online meeting platforms, the technology’s adoption to support breastfeeding is spotty. Technology-based prenatal breastfeeding education should be encouraged, especially during the pandemic. Prompt and effective adoption of online meeting platforms to help provide education and training during the prenatal and postpartum period will help prepare for successful and longer breastfeeding infants.

### 3.6. Evolution of the Lactation Consultant

The services of antenatal counseling, breastfeeding education, and help with practical aspects of breastfeeding the infant after birth should be a team-based approach with the lactation consultant at the team’s core. The deficiencies of breastfeeding support have been laid bare during the pandemic. Revamping the breastfeeding support structure to meet pregnant and lactating mothers’ needs is a priority. Technological advances could be used for the seamless delivery of breastfeeding education and support. Integration of services with one team, including lactation specialists, would help seamless service continuation, avoid duplication of services, and economize resources. There is an opportunity here to standardize breastfeeding education in a way to reach all expectant families. The standardization of breastfeeding education should be across all platforms, including online training, smartphone applications, and in-person training whenever possible. Mothers should have the choice to decide on appropriate media of training and support depending on their circumstances.

## 4. Unanswered Questions and Further Research

There is an imminent need for data on the safety of COVID vaccines during pregnancy and lactation. The Center for Disease Control monitors data through their smartphone app, V-safe [33], a tool that uses text messaging and web-based surveys to provide personalized health check-ins and where vaccine recipients can report side effects following vaccination for COVID-19. The preliminary findings did not show obvious safety signals among pregnant persons who received mRNA COVID-19 vaccines in the third trimester [37]. However, more longitudinal follow-up, including follow-up of large numbers of women vaccinated earlier in pregnancy, is necessary to inform maternal, pregnancy, and infant outcomes.

Even at the best of times, most women who say they want to breastfeed either are not able to or run into challenges that force them to stop sooner than they had hoped. The pandemic has worsened the situation. Promoting breastfeeding and helping new mothers in becoming confident to breastfeed are becoming a challenge. Challenges to providing online help with lactation consultants and how to facilitate face-to-face support to resolve issues that require one, as well as challenges supporting moms both in the hospitals that are understaffed and in the community, are still unanswered questions.

Breastfeeding a newborn baby is one of the fundamental necessities for the healthy survival of humans. Breastmilk nutrition affects every organ of the baby and the mother, as well as the infant’s overall physical, mental, and emotional health. The long-term benefits of breastfeeding are starting to be understood. It is time for us to put in more resources and use technological advances to find innovative solutions at the community and the individual level to facilitate mother–infant bonding soon after birth. The pandemic broke the support structure concerning breastfeeding. We must build a robust breastfeeding support structure both in the prenatal and the postnatal period, with lactation consultants at the core of this support structure. Lactation consultants should be regarded as practitioners to independently bill and get reimbursed for the work they do. Promotion and optimal utilization of lactation consultants will go a long way in building a healthy society of the future.

There is a need for breast milk testing on a larger scale of those who received COVID vaccines to demonstrate the presence of antibodies and, eventually, its long-term effect on avoiding severe infections in newborns and infants. As vaccine uptake increases in the following months, answers should be forthcoming regarding its efficacy in protecting infants from disease. Data on vertical transmission and antibody transfer into breast milk should be more precise. The time for rigorous research and to find innovative solutions for all the problems encountered during the pandemic is now so that we can prepare ourselves for the future.

## 5. Conclusions

Breast milk is scientifically proven to be best for newborn children and providing it through breastfeeding is one of the most basic and loving acts desired by most mothers. We need a collective effort from the scientific community to address unanswered questions relating to COVID-19. Additionally, intelligent health care policies that provide paid leave and breastfeeding support are vital for healthy mother–infant bonding. The benefits of an appropriate start soon after birth are enormous in meeting healthy child development goals. Advances in technology should be applied to create innovative solutions for the challenges posed by the pandemic. An ‘all-in’ comprehensive approach wherein the goal is to support pregnant and lactating mothers is a priority and indispensable for our shared future.

## Figures and Tables

**Table 1:** Innovative solutions to facilitate breastfeeding.

Supporting Breastfeeding in Year 2021 and Beyond
Supportive Legislations Paid Parental Leave Supportive breastfeeding baws with enforcement and penalty powers.
Innovative solutions to support breastfeeding Embracing technology to standardize breastfeeding education for all expecting parents and lactating mothers. Connecting their mothers to their separated babies admitted in hospital. Innovation equity and equality.
Pairing proactive virus surveillance with breastfeeding research.
Combating misinformation.

## Data Availability

Not applicable.
